# Catalytic Oxidation of Phenol and 2,4-Dichlorophenol by Using Horseradish Peroxidase Immobilized on Graphene Oxide/Fe_3_O_4_

**DOI:** 10.3390/molecules21081044

**Published:** 2016-08-10

**Authors:** Qing Chang, Jia Huang, Yaobin Ding, Heqing Tang

**Affiliations:** College of Resources and Environmental Science, South Central University for Nationalities, Wuhan 430074, China; changqinghust@163.com (Q.C.); jjhhhust@163.com (J.H.); dyaobin@126.com (Y.D.)

**Keywords:** graphene oxide, magnetite, phenol, immobilized enzyme, catalytic oxidation

## Abstract

Graphene oxide/Fe_3_O_4_ (GO/Fe_3_O_4_) nanoparticles were synthesized by an ultrasonic-assisted reverse co-precipitation method, and then horseradish peroxidase (HRP) was covalently immobilized onto GO/Fe_3_O_4_ with 1-ethyl-3-(3-dimethyaminopropyl)carbodiimide (EDC) as a cross-linking agent. In order to enhance the phenol removal efficiency and prevent the inactivation of the enzyme, the polyethylene glycol with highly hydrophilicity was added in this reaction, because the adsorption capacity for the polymer by degradation was stronger than the HRP. The results showed that the immobilized enzyme removed over 95% of phenol from aqueous solution. The catalytic condition was extensively optimized among the range of pH, mass ratio of PEG/phenol as well as initial concentration of immobilized enzyme and H_2_O_2_. The HRP immobilized on GO/Fe_3_O_4_ composite could be easily separated under a magnetic field from the reaction solution and reused.

## 1. Introduction

A great amount of industrial wastewaters containing phenolic compounds are generated from coal conversion, resins and plastics processing, coke combustion, and so on. Many phenolic compounds are a potential danger to human health and suspected to be carcinogen [1,2]. One treatment approach is based on the oxidation of phenolic pollutant by natural enzymes [3]. This treatment has many advantages compared with other biological or chemical/physical methods. It is easier than microorganism manipulation for handling and storage of isolated enzymes. Moreover, the specificity of enzymes is much higher with respect to other conventional catalysts.

Horseradish peroxidase (HRP) is a classic heme enzyme containing a ferric protoporphyrin IX prosthetic group. In the presence of hydrogen peroxide (H_2_O_2_), HRP catalyzed the oxidation of phenols to form phenoxy radicals [4]. The free radical products spontaneously formed insoluble high molecular weight polymers, which could be removed from solution by filtration or sedimentation [5]. However, during the enzymatic treatment, the generated phenoxy radicals may interact with the active center of the enzyme, leading to shortened catalytic lifetime of the enzyme [6]. In addition, the generated polymers may entrap HRP, decreasing its catalytic ability. In order to overcome the problem of the inactivation of the enzyme, it was reported that the enzyme lifetime was significantly improved by using polyethylene glycol (PEG) or gelatin with high hydrophilicity, which have a greater affinity than HRP for the hydroxyl groups on the growing polymers [7,8]. Another strategy for overcoming the problem of the inactivation of the enzyme was to immobilize enzymes.

It is demonstrated that enzyme immobilization can endow enzymes with some additional advantageous properties [9]. Compared with free enzyme, immobilized enzymes dispersed on the support surface will suffer from less aggregation, which is another inactivation source [10]. A proper enzyme immobilization may produce a strong rigidification of the enzyme structure, especially if a very intense multipoint covalent attachment of enzymes on the support via short spacer arms is achieved [11]. Because all enzyme residues involved in the enzyme immobilization process should maintain their relative positions during any conformational change induced by more drastic conditions, it reduced any conformational change involved in enzyme inactivation and significantly improved the enzyme stability [12,13]. Besides the immobilized enzymes exhibit higher selectiveness and specificity [14]. Nonetheless, enzymes can be denatured and lose their activity simply when solubilized in the immobilization medium and during the immobilization procedure. The immobilization of enzymes may block the active centers and promote diffusion problems [10]. It is also reported that the diffusional limitation (e.g., the generation of gradients of pH) in the porous support immobilized with very active enzyme might provide a profitable tool to improve the operational parameters of enzymes [15]. It is also suggested that the immobilized enzymes can be used repeatedly or continuously in a variety of reactors for the efficient recovery of costly enzymes, and be easily separated from reaction systems for reuse, which make the work-up simple and the protein of the final product uncontaminated [16].

Nanoparticles are considered to be excellent supports for enzyme immobilization because of their minimized diffusional limitations, high specific surface area and high enzyme loading capability, mechanical stability, which are interesting and important features for the immobilization process [17]. Nevertheless, the purity of the enzyme sample only impairs the volumetric activity when using non-porous nanoparticle supports [10]. Moreover, the recovery by centrifugation and filtration of these nanoparticles is very difficult. Magnetic supports show the obvious feature of being ferromagnetic, which allows recovery of the immobilized enzyme preparation from a reactor by simply applying a magnetic field to the slurry, without the need of tedious decantation, centrifugation, or filtration procedures [18]. Fe_3_O_4_ magnetic nanoparticles (MNPs) are known to be biocompatible, display no hemolytic activity and genotoxicity with superparamagnetic properties and have been utilized in various fields [19]. It was found that Fe_3_O_4_ MNPs have the peroxidase-like property and could activate H_2_O_2_ to degrade organic pollutant [20]. Furthermore, Fe_3_O_4_ MNPs can feasibly combine with easily modifiable materials, and modify inherently their surface with functional groups to link interested enzymes. Graphene oxide (GO), a derivation of graphene, having a large specific surface area and a range of reactive oxygen functional groups on the surface, not only renders it a good candidate for supporting metal or metal oxide particles, but also provides an ideal substrate for enzyme immobilization [21]. In this study, GO/Fe_3_O_4_ MNPs were synthesized with an ultrasonic-assisted reverse co-precipitation method, and then HRP was covalently immobilized onto the GO/Fe_3_O_4_ as an enzyme carrier. This composite system could highly efficiently catalyze the reduction of H_2_O_2_ to remove phenol and 2,4-dichlorophenol (2,4-DCP).

## 2. Results and Discussion

### 2.1. Characterization of Materials

**Figure 1:**
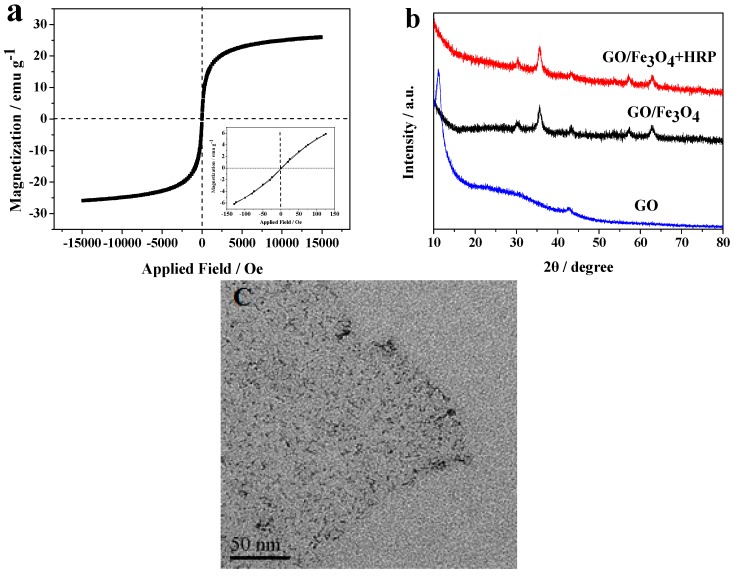
(**a**) Magnetization curve of GO/Fe_3_O_4_; (**b**) The X-ray diffraction patterns of GO, GO/Fe_3_O_4_ and GO/Fe_3_O_4_ + HRP; (**c**) TEM image of GO/Fe_3_O_4_ + HRP.

The magnetization curve of GO/Fe_3_O_4_ nanoparticles was measured at room temperature, as shown in Figure 1a, which demonstrated that the GO/Fe_3_O_4_ exhibited negligible coercivity (1.17 Oe) and remanence (73.06 × 10^−3^ emu/g), being typical of superparamagnetic materials. The specific saturation magnetization, *M*_s_ of the sample, was 25.96 emu/g. This value was smaller than the reported value of bulk Fe_3_O_4_ of 68.80 emu·g^−1^ [19]. Fe_3_O_4_ was estimated as 37.7 wt % calculated from the content of GO/Fe_3_O_4_. X-ray powder diffraction was used to identify the produced powders as shown in Figure 1b. The broad small characteristic peak of GO around 24° can be observed, which was attributed to C(002) reflection of graphite oxide based on the short-range order in stacked sheets [22]. The diffraction peaks at 11.0° was assigned to the characteristic GO (001) crystal. The characteristic diffraction peaks (2θ) of GO/Fe_3_O_4_ at 30.4, 35.6, 43.3, 53.0, 57.1, and 62.8° were ascribed to the (220), (311), (400), (422), (511) and (440) planes of Fe_3_O_4_, being similar to the previously reported data [23,24]. For magnetic particles with bound HRP, the same six characteristic peaks were observed. This suggested that the binding process did not result in the phase change of GO/Fe_3_O_4_. As a result, the magnetic particles could preserve their magnetic properties during the separation process, which was suitable for application in bioseparation. A TEM image of the prepared immobilized enzyme indicated GO sheets uniformly decorated with nanoparticles with a narrow size distribution, as shown in Figure 1c. The morphology is similar to GO/Fe_3_O_4_ nanoparticles synthesized in our previous work [21].

### 2.2. Reaction System and Effect of PEG

**Scheme 1:**
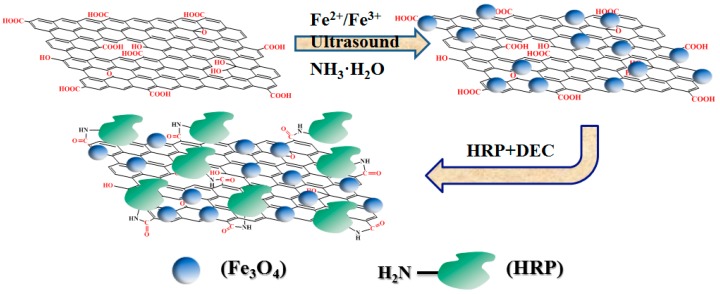
Schematic illustration of HRP immobilized on GO/Fe_3_O_4_ by EDC.

The procedures for the synthesis of GO/Fe_3_O_4_ particles and the immobilization of HRP onto GO/Fe_3_O_4_ with EDC as a cross-linking agent are illustrated in Scheme 1. The amount of immobilized enzyme was about 5.3 mg/g on the magnetic GO/Fe_3_O_4_ nanoparticles. The initial HRP specific activity was 128 U/mg and the measured activity of the immobilized HRP was about 440 U/g, while retained about 65% of initial HRP activity.

**Figure 2:**
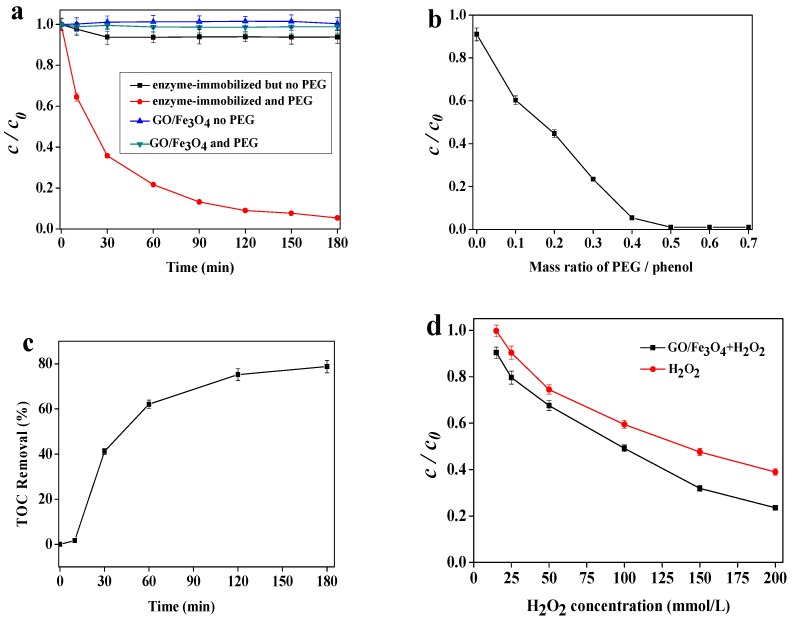
(**a**) The removals of phenol in different systems; (**b**) Effect of mass ratio of PEG/phenol dose on phenol removal; (**c**) The time dependence of removal TOC; (**d**) Effect of H_2_O_2_ concentration on phenol removal in Fe_3_O_4_/GO system without HRP.

As it was known, inactivation is due to the adsorption of enzyme onto the polymers formed during the reaction [8]. In order to reduce the amount of the enzyme, several researchers have suggested that highly hydrophilic additives such as PEG and gelatin can be used to protect the enzyme from inactivation during phenol removal. In Figure 2a, when there is an absence of PEG, the phenol removal efficiency is only about 9%. Within a 3-h reaction period, the removal of phenol achieved 95% in the GO/Fe_3_O_4_-HRP system, being much greater than the GO/Fe_3_O_4_ and GO/Fe_3_O_4_ + PEG system (almost no reduction). Correspondingly, in Figure 2c, about 78% of the total organic carbon (TOC) was found to be removed. As we all know, Fe_3_O_4_ and GO are proved that possess enzyme mimetic activity [20,21]. However, the phenol was not removed, because the amount of the H_2_O_2_ was fairly small compared to other reports [20].

As shown in Figure 2b, before the mass ratio of PEG/phenol reaches 0.4, they are significantly enhanced with the increase of the amount of PEG added. The further addition of PEG would result in slightly reduction of the phenol removal efficiency, which was in agreement with the result reported by Cheng et al. [25]. Moreover, the phenol removal efficiency was over 95% at this time. Both GO and Fe_3_O_4_ possess intrinsic peroxidase-like activity, and we studied the activity of the GO/Fe_3_O_4_. The concentration of H_2_O_2_ changed when there is only GO/Fe_3_O_4_. As the amount of H_2_O_2_ is increased, the phenol removal grows in quantity (Figure 2d). It is evident from the Figure 2d that about 76% of the phenol is found to be removed because of the GO/Fe_3_O_4_ catalyzed reaction.

### 2.3. Effect of the Immobilized Enzyme Dose

**Figure 3:**
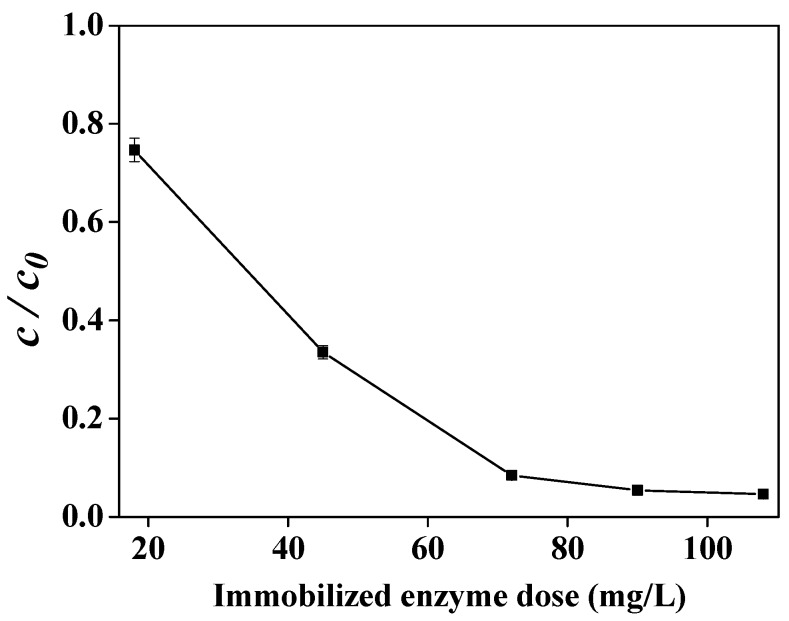
Effect of immobilized HRP dose on phenol removal.

The catalyst effect of the immobilized enzyme dose is studied, as shown in Figure 3. As the amount of the immobilized enzyme is increased, the phenol removal is significantly promoted until the concentration is about 90 mg/L. The increase of the phenol removal slowed when the dose of immobilized enzyme is higher than 90 mg/L. With HRP concentration increase, more inert intermediate product, phenoxyl radicals, which increase the inactivation chance, will be produced. Therefore, the load of immobilized enzyme was optimized at 90 mg/L.

### 2.4. Effect of H_2_O_2_ Concentration

**Figure 4:**
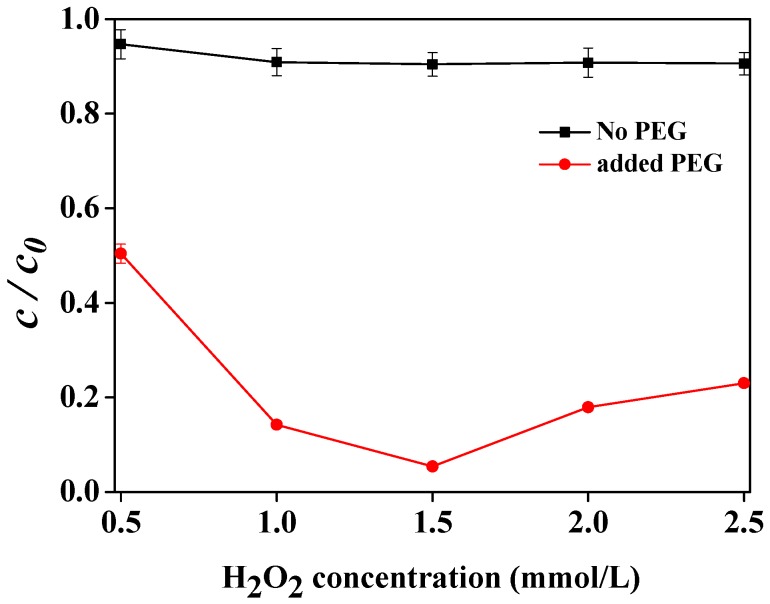
Effect of H_2_O_2_ concentration on phenol removal in the absence and presence of PEG.

According to the experimental results in the Figure 4, a conclusion can be made that there is a relationship between total phenols removed and a function of H_2_O_2_ provided during the experiment when the molar ratio of H_2_O_2_ to phenol is 1.5, regardless of PEG, which is the optimal conditions in this experiment. Some others reported that the optimum molar ratio of H_2_O_2_ to phenol was 1.0 or 2.0, which might result from polymers larger than dimmers being produced during the catalytic process [26,27]. Enzymes can be inactivated by too much hydrogen peroxide.

### 2.5. Degradation of Phenol and 2,4-Dichlorophenol in the Mixture

**Figure 5:**
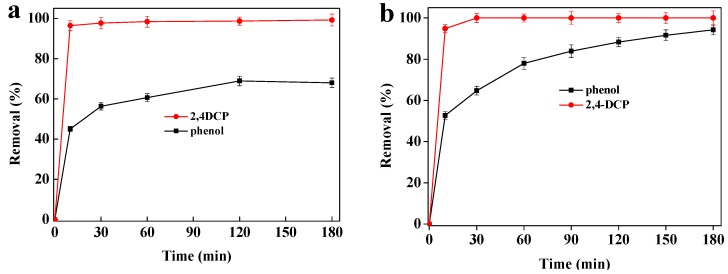
Time profiles of the removals of phenol and 2,4-DCP during the oxidation of: (**a**) phenol and 2,4-DCP alone; and (**b**) their mixture.

As shown in Figure 5a, 2,4-dichlorophenol has almost been completely removed totally, but 30% of phenol remains. When phenol and 2,4-dichlorophenol are mixed, about 94% of the phenol was found to be removed, similar to the amount previously reported [28], which mean that the easily removed compound (2,4-dichlorophenol) can facilitate the removal of the hard one (phenol). This phenomenon is probably due to the different phenoxy radicals produced by the enzyme. The radicals produced from easy-to-remove phenols can react with the hard-to-remove ones. Therefore, phenol can be removed more easily in presence of 2,4-dichlorophenol than individual one.

### 2.6. Reusability

**Figure 6:**
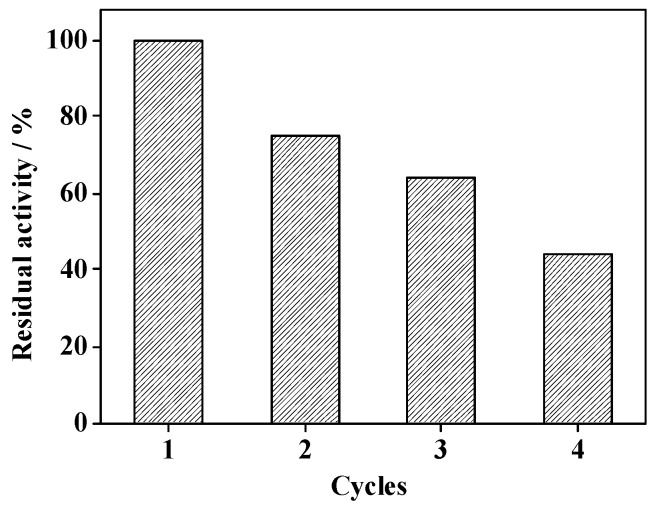
Reusability of the immobilized enzyme in phenol removal.

In practical application one attractive advantage of the immobilized enzyme is that it can be easily separated from the reaction system and reused, which greatly decreases the cost of the enzyme. The efficiency of phenol removal reduced with the cycle of the immobilized enzyme as shown in Figure 6. After the first cycle of the catalytic reaction with the HRP immobilized on GO/Fe_3_O_4_, the immobilized enzyme was recovered by centrifuging in order to remove the supernatant, and the immobilized HRP was repeatedly washed with double distilled water and the activity was assayed again. After four cycles, enzyme activity dropped to 40% of its initial activity. The reusability of the immobilized enzyme is dependent on solid support. The activity decline of the immobilized HRP on GO/Fe_3_O_4_ can probably be attributed to the polymers produced during the enzymatic reaction, which may cover the enzyme and affect the reaction of the next cycle. Further investigations on improvement of reusability and application of the immobilized enzyme in a continuous-flow device are in process.

## 3. Experimental Section

### 3.1. Reagents and Materials

HRP was purchased from Tianyuan Biologic Engineering Corp. (Wuhan, China) and stored at −20 °C. FeCl_3_·6H_2_O, FeSO_4_·7H_2_O, NH_3_·H_2_O, H_2_O_2_ (30%, *w*/*w*), phenol, 2,4-DCP, EDC, PEG, NaH_2_PO_4_·2H_2_O, Na_2_HPO_4_·12H_2_O, potassium ferricyanide and 4-aminoantipine (4-AAP) were purchased from Sinopharm Chemical Reagent Co, Ltd. (Shanghai, China). All the chemical reagents were of analytical grade and used without further purification. The water used was double distilled water.

### 3.2. Preparation of GO/Fe_3_O_4_ Nanoparticles

Oxidized graphite was prepared from graphite powder through a modified Hummers method [29]. The GO/Fe_3_O_4_ nanoparticles were synthesized with an ultrasonic-assisted reverse co-precipitation method [30]. In a typical experiment, 0.2 g GO (water suspension), FeSO_4_·7H_2_O (0.48 g) and FeCl_3_·6H_2_O (0.466 g) were dissolved in 50 mL of distilled water. The mixed solution was added dropwise into 20 mL of distilled water in which 3 mL of ammonia water (30%) was dissolved. The reaction was carried out at 60 °C in an ultrasound clean bath operating at 25 kHz with a power of 140 W (KQ-200KDE, Kunshan, China). After 60 min for the reaction, the generated black GO/Fe_3_O_4_ nanoparticles were collected by magnetic separation, washed with water to neutral pH, and then dried under vacuum at 50 °C.

### 3.3. Immobilization of HRP on GO/Fe_3_O_4_

The above obtained GO/Fe_3_O_4_ (0.2 g) was dispersed in 20 mL of PBS solution (pH 6.0) containing 1 mg HRP. One milliliter of EDC (25 mg/L) was added into the mixture. The immobilization was carried out with shaking for 12 h at 25 °C. The nanoparticles were separated and washed with PBS solution and water to remove the free enzyme. The enzyme-immobilized nanoparticles were then redispersed in PBS (pH 7.0) solution and stored at 4 °C.

### 3.4. Characterization

UV-visible absorption spectra were recorded on a Cary 50 UV-Vis spectrophotometer (Varian, Palo Alto, CA, USA). The magnetic properties were performed using ADE 4HF vibrating sample magnetometer (Lowell, MA, USA) at 300 K. X-ray diffraction patterns were recorded on an X’Pert PRO X-ray diffractometer (PANalytical, Almelo, The Netherlands) with a Cu Ka radiation source generated at 40 kV and 30 mA.

### 3.5. Enzyme Activity Measurements

The colorimetric procedure was used to determine the activity of enzyme [8], in which phenol, 4-AAP and H_2_O_2_ were used as substrates. A mixture was obtained by adding 0.1 mL 0.2 mol/L phenol, PBS (pH 7.4), 0.1 mL 4.8 × 10^−2^ mol/L 4-AAP, 20–100 μL solution of free or immobilized HRP and 20 μL 2 × 10^−3^ mol/L H_2_O_2_ into a cuvette. The total volume was adjusted by water to 1.8 mL. The catalytic reaction was monitored by recording the absorbance of its red product at 510 nm. One unit of the activity (U) of HRP was defined as the amount of HRP required to hydrolyze 1 × 10^−6^ mol of H_2_O_2_ converted per minute under the conditions stated above.

### 3.6. Experiments for Removing Phenol and the Mixture of Phenol and 2,4-Dichlorophenol

The experiment for removing phenol was carried out in 100 mL conical flasks in a temperature-controlled water bath shaker (SHZ-82A) at temperature of 25 °C and a constant shaking speed of 160 rpm. The typical concentration in the reaction solution (pH 7.4) contained phenol (1 mmol/L), immobilized enzyme (90 mg/L), PBS (25 mmol/L) and PEG (the mass ratio of PEG/phenol was set at 0.4). After 15 min, to achieve of adsorption–desorption equilibrium of phenol on the GO/Fe_3_O_4_ particles, the degradation was initiated by rapid adding H_2_O_2_ (1.5 mmol/L) to the reaction solution, the total volume was rapidly adjusted to 40 mL with distilled water. At given time intervals, 2 mL aliquots of the reaction solution were sampled, and immediately centrifuged at 14,000 rpm for 3 min with an EBA-21 centrifugal (Hettich, Germany) to remove any sediment.

The experiment for removing the mixture of phenol and 2,4-dichlorophenol was carried out under almost the same conditions as described for removing the mixture of phenol. The difference was that a mixture of phenol (0.5 mmol/L) and 2,4-dichlorophenol (0.5 mmol/L) was used instead of phenol alone, and the load of PEG was 42 mg/L. Each run of the experiments was replicated at least three times.

The detection of the concentrations of phenol and 2,4-dichlorophenol were conducted on high performance liquid chromatography (U-3000 HPLC, Thermo scientific, Waltham, MA, USA) equipped with a C-18 column (5 μm,150 × 4 mm). The mobile phase was methanol/water (60/40) at a flow rate of 0.8 mL/min and detection wavelength was 270 nm for phenol and 288 nm for 2,4-dichlorophenol. Total organic carbon was measured by a TOC (Vario TOC SELECT, Elementar, Hanau, Germany).

## 4. Conclusions

In this study, GO/Fe_3_O_4_ nanoparticles were synthesized by an ultrasonic-assisted reverse co-precipitation method that was fast and simple, compared with others reported. HRP was successfully immobilized on the GO/Fe_3_O_4_ via interaction between functional groups of GO/Fe_3_O_4_ and HRP with EDC. We investigated the catalytic property in phenolic compound removal. Compared with soluble HRP, HRP immobilized on GO/Fe_3_O_4_ exhibited better thermal stability. PEG improved the enzymatic treatment efficiency of synthetic wastewater significantly. However, the reusability of the immobilized HRP was not satisfactory, possibly because polymers generated during reaction adsorption on the enzyme surface led to enzyme reduction during the reaction cycle. Further investigations on improvement of reusability and application of the immobilized enzyme are in process.
